# Cholinesterase inhibitors and memantine are associated with a reduced mortality in nursing home residents with dementia: a longitudinal observational study

**DOI:** 10.1186/s13195-024-01481-0

**Published:** 2024-05-29

**Authors:** Charlotte Havreng-Théry, Bruno Oquendo, Victoria Zolnowski-Kolp, Pierre Krolak-Salmon, François Bertin-Hugault, Carmelo Lafuente-Lafuente, Joël Belmin

**Affiliations:** 1 Laboratoire LIMICS, Sorbonne Université, Paris, France; 2 Service de Gériatrie, Hôpital Charles Foix, Ivry-sur-Seine, 94200 France; 3 Présage Care, Paris, France; 4 Groupe Orpéa, Puteaux, France; 5 Laboratoire CEPIA, Université Paris Est, Créteil, France

**Keywords:** Cholinesterase inhibitors, Memantine, Nursing home, Long term care facilities, Mortality

## Abstract

**Background:**

A large proportion of nursing home (NH) residents suffer from dementia and effects of conventional anti-dementia drugs on their health is poorly known. We aimed to investigate the associations between exposure to anti-dementia drugs and mortality among NH residents.

**Methods:**

This retrospective longitudinal observational study involved 229 French NH and the residents admitted in these facilities since 2014 and having major neurocognitive disorder. From their electronic health records, we obtained their age, sex, level of dependency, Charlson comorbidity index, and Mini mental examination score at admission. Exposure to anti-dementia drugs was determined using their prescription into 4 categories: none, exposure to acetylcholinesterase inhibitors (AChEI) alone, exposure to memantine alone, exposure to AChEI and memantine. Survival until the end of 2019 was studied in the entire cohort by Cox proportional hazards. To alleviate bias related to prescription of anti-dementia drugs, we formed propensity-score matched cohorts for each type of anti-dementia drug exposure, and studied survival by the same method.

**Results:**

We studied 25,358 NH residents with major neurocognitive disorder. Their age at admission was 87.1 + 7.1 years and 69.8% of them were women. Exposure to anti-dementia drugs occurred in 2,550 (10.1%) for AChEI alone, in 2,055 (8.1%) for memantine alone, in 460 (0.2%) for AChEI plus memantine, whereas 20,293 (80.0%) had no exposure to anti-dementia drugs. Adjusted hazard ratios for mortality were significantly reduced for these three groups exposed to anti-dementia drugs, as compared to reference group: HR: 0.826, 95%CI 0.769 to 0.888 for AChEI; 0.857, 95%CI 0.795 to 0.923 for memantine; 0.742, 95%CI 0.640 to 0.861 for AChEI plus memantine. Results were consistent in propensity-score matched cohorts.

**Conclusion:**

The use of conventional anti-dementia drugs is associated with a lower mortality in nursing home residents with dementia and should be widely used in this population.

**Supplementary Information:**

The online version contains supplementary material available at 10.1186/s13195-024-01481-0.

## Background

Major neurocognitive disorders (M-NCDs) also known as dementia are widespread conditions, affecting around 50 million people worldwide, and Alzheimer’s disease is the most common form of dementia, accounting for 60–70% of cases [1]. With advancing age, the most important risk factor, the number of people living with these diseases is set to rise sharply as the world’s population ages, and by 2050, the prevalence of M-NCDs is expected to triple. Alzheimer’s disease and other M-NCDs are responsible for a global decline in cognitive functions, including memory and in independence, requiring human assistance for activities of daily living, and frequently they lead to behavioural disorders [1]. All these features are common reasons for admission to nursing homes, and it is estimated that around two-thirds of nursing home residents are affected by neurocognitive diseases. In addition, Alzheimer’s disease and other M-NCDs are responsible for increased mortality, and a recent meta-analysis revealed that all-cause mortality was multiplied by 5.9 in patients with M-NCDs compared to age-matched individuals without these diseases [1–3].

Only a few pharmacological treatments are currently available for M-NCD, and their indication depends on the subtype. No pharmacological treatment is indicated for vascular dementia or frontotemporal dementia. Acetylcholinesterase inhibitors (AChEI) are indicated in M-NCD due to Alzheimer’s disease, Lewy body disease and Parkinson’s disease, and memantine is indicated in M-NCD due to Alzheimer’s disease. These drugs have been marketed in the USA and Europe for over 20 years, and the value of these molecules is controversial, although their efficacy has been demonstrated by well-conducted randomized trials [4, 5]. In fact, their effects on the decline of cognition and independence are modest, and randomized trials did not show any reduction in mortality [4–8]. In addition, certain side-effects that came to light after they were launched on the market have also contributed to their being called into question. More recently, the anti-amyloid antibodies aducanumab and lecanemab have been approved in the USA but not in Europe, and their clinical value is also controversial due to their very high price and moderate efficacy/tolerance profile [9, 10]. These molecules have also shown no effect on patient mortality.

Conventional anti-dementia drugs have interesting biological properties that could have favourable effects not only on neurons, but also on the function of other human cells and on the cardiovascular system. AChEIs have vagotonic effects and slow the heart rate [11], while memantine protects cells from excitotoxicity by antagonising NMDA receptors [12]. AChEI and memantine also have antioxidant and anti-inflammatory properties [11, 12]. This has stimulated observational studies to determine whether the use of anti-dementia drugs is associated with a reduction in dementia mortality. In fact, although individual randomised trials have not shown such effects, the size of these trials and the relatively short follow-up times have not allowed an in-depth investigation of these possible effects. Very few of these studies have examined the relationship between mortality and the use of anti-dementia drugs in the specific context of nursing homes, despite the fact that a very large number of older adults with dementia live in these facilities. From our point of view, the studies carried out in this context are interesting because they concern very old, complex and vulnerable people, who are poorly represented in research studies, and the results obtained can guide our practices not only for nursing home residents, but also for older adults living at home who have a comparable profile.

The effects of conventional anti-dementia drugs on patient mortality have been the subject of studies with varying results [13, 14]. In this context, our team carried out a meta-analysis to explore the AChEI on mortality of people with dementia that showed that AChEI were associated with a significant reduction in mortality in both randomised trials and observational studies, with a risk reduction of around 15% [15]. Of the 24 studies included in this review, only one had been conducted in nursing homes, and their authors found that the 5423 residents which received donepezil had a significantly lower mortality than 5423 matched residents of the same facilities that did not received anti-dementia drugs (hazard ratio: 0.90; 95% CI, 0.84–0.96) [14]. Studies that investigated association of memantine with mortality are rare and no evidence is available for nursing home residents.

The aim of this study was to investigate the associations between mortality and exposure to anti-dementia drugs in nursing home residents with dementia.

## Methods

### Design, participants and data source

This retrospective observational cohort study has been conducted on the residents admitted after January 1st, 2014 in the 229 nursing homes of a French group of private nursing homes. These facilities were located in all regions of France in both urban and rural areas. De-identified data until December 31st, 2019 were obtained from their electronic health record (EHR). Their EHR was filled by residents’ general practitioners and by the medical and paramedical staff of the nursing home. We included in the analysis all residents with an explicit diagnosis of Alzheimer’s disease, dementia or M-NCD mentioned in the EHR and those who received donepezil, rivastigmine, galantamine or memantine. In addition, we included residents with overt and prolonged cognitive impairment defined by a Mini Mental Status Examination (MMSE) score < 20 on at least two separate occasions. All were presumed to have M-NCD. The flowchart that describes the constitution of the cohort is shown in the supplementary material (Figure S1). We retrieved from EHR residents’ age at admission, gender and the level of dependency assessed by the Grille AGGIR, the French national scale used for resources allocation to disabled adults > 60 years in France. The scale is based on the rating of 17 variables describing activities of daily living and each variable is quoted on 3 levels according the ability of the person to perform the activity him/herself without human assistance. Based on these ratings, an algorithm designed to estimate the amount of human assistance required for activities of daily living, allocates the person to one of six groups (groupes iso-ressources or GIR): GIR 1 to GIR 6. The group GIR1 corresponding to person with the most severe dependency and who require the highest level of assistance, and group GIR 6 to persons with no dependency who require little or no human assistance. We also recorded the first MMSE score notified in the EHR, and calculated the Charlson comorbidity index from the diseases notified in the EHR and age at admission.

### Exposure to anti-dementia drugs and outcomes

Information about anti-dementia prescription was obtained from orders directly entered into the EHR by the residents’ general practitioner. Exposure to AChEI was defined by any prescription of donepezil, rivastigmine or galantamine, irrespective of their duration. Exposure to memantine or exposure to AChEI plus memantine was defined similarly.

Outcome was the mortality studied from the vital status recorded until December 31st, 2019 and primary criteria of judgment were the adjusted mortality hazards ratio in matched cohorts.

### Statistics

Four groups were formed according to exposure to anti-dementia drugs: None (neither CEI or memantine), AChEI only, Memantine only, and both AChEI and memantine. Their characteristics were compared using one-way ANOVA, the chi-2 test and the Kruskal Wallis test for the variables that were not normally distributed.

Unadjusted and adjusted mortality hazard ratios and their 95% confidence intervals (95%CI) were calculated using Cox proportional hazards models for these four groups using non exposed group as the reference. For adjusted HRs, the controlled covariates were age, sex, level of dependency, MMSE score and Charlson index (model 1) or adjusted on age, sex, level of dependency, MMSE score and individual comorbidities variables (model 2). Survival curves were drawn using Kaplan-Meier method and the log-rank test was used to determine whether survival curves differed statistically. The hazard ratio for the combined therapy (AChEI plus memantine) has been compared to those of AChEI alone or to memantine alone using the Wald test.

To control for treatment indication bias, we have also studied association between mortality and each anti-dementia group in three propensity score–matched cohorts. First, residents exposed to AChEI alone were matched with residents not exposed to anti-dementia drugs (matched cohort 1) on their propensity score by nearest neighbor method using two neighbors for one case, within a caliper of 0.005 SD. Variables used to define treatment allocation were age at baseline, sex, first MMSE score, the level of dependency and the Charlson index values. The quality of the propensity score–matched cohorts was assessed with the standardized mean difference. Two other matched cohorts were elaborated using the same methods, one for the residents exposed to memantine alone (matched cohort 2) and another for residents exposed to AChEI plus memantine (matched cohort 3). A standardized mean difference greater than 0.20 was considered a sign of imbalance. For each matched cohort, we have plotted survival curves using Kaplan Meier method and we used using Cox proportional hazards models to calculate mortality hazard ratios and their 95% confidence intervals adjusted on age, sex, level of dependency, MMSE score and Charlson index (model 1) or adjusted on age, sex, level of dependency, MMSE score and individual comorbidities variables (model 2).

Calculations were computed using Stata software 16.1 (Stata Corps, USA) and the level of significance was *P* < 0.05.

## Results

### Entire cohort

Data from the EHR of 45,606 residents were available in the period considered and 25,358 (55.6%) were selected for the study according the flowchart shown in supplementary material (Supplementary material, Figure S1). Age at admission was 87.1 + 7.1 years and they comprised 7,647 (30.1%) men and 17,711 (69.8%) women. Mean follow-up was 18.8 months and there was no loss of follow-up. Their characteristic according the exposure to anti-dementia drugs are shown in Table 1. Exposure to anti-dementia drugs occurred in 5065 residents (20.0%) comprising 2,550 (10.1%) for AChEI alone, in 2,055 (8.1%) for memantine alone, in 460 (0.2%) for AChEI plus memantine, whereas 20,293 (80.0%) had no exposure to anti-dementia drugs. Anti-dementia drugs prescription was present on admission in 87% of them and median duration of exposure was 7.1 months (IQR: 2.8, 17.5). Exposure of less than 30 days was observed in 664 residents (13.1%). The residents not exposed to anti-dementia drugs were significantly older, comprised a greater proportion of women and their level of dependency and their cognitive impairment were less severe. The MMSE score of the residents exposed to both AChEI and memantine was significantly higher than those of the other groups (Table 1). Severe dependency was significantly more frequent in residents exposed to memantine only or to the association AChEI plus memantine. Some comorbidities like cardiovascular disease, diabetes and chronic obstructive lung disease were more frequent in residents unexposed to anti-dementia drugs.

**Table 1:** Characteristics of nursing home residents with dementia (entire cohort) according to their exposure to cholinesterase inhibitors (AChEI), memantine, and AChEI plus memantine

	Exposure to anti-dementia drugs				*P*
	None	AChEI	Memantine	AChEI + memantine	
	(*n* = 20,293)	(*n* = 2,550)	(*n* = 2,055)	(*n* = 460)	
Age in years, mean (sd)	87.7 (6.9)	84.3 (6.7)	85.3 (6.3)	83.6 (6.6)	< 0.001
Males (n,%)	5,957 (29.35)	858 (33.65)	676 (32.9)	156 (33.91)	< 0.001
Level of dependency (n,%)					< 0.001
very severe (GIR1)	853 (4.42)	148 (6.39)	169 (9.06)	39 (9.29)	
severe (GIR2)	6,553 (33.96)	938 (40.48)	794 (42.57)	198 (47.14)	
moderately severe (GIR3)	4,552 (23.59)	510 (22.01)	435 (23.32)	78 (18.57)	
moderate (GIR4)	5,600 (29.02)	565 (24.38)	364 (19.52)	83 (19.76)	
mild (GIR5)	1,090 (5.65)	93 (4.01)	73 (3.91)	13 (3.10)	
none (GIR6)	648 (3.36)	63 (2.72)	30 (1.61)	9 (2.14)	
MMSE score, m (sd)	16.0 (7.3)	17.2 (8.6)	16.0 (9.5)	15.5 (9.6)	< 0.001
Cardiovascular diseases (n,%)	7,355 (36.24)	676 (26.51)	659 (32.01)	122 (26.52)	< 0.001
Peripheral arterial disease (n,%)	603 (2.97)	46 (1.80)	53 (2.58)	6 (1.30)	< 0.001
Stroke (n,%)	630 (3.10)	61 (2.39)	53 (2.58)	16 (3.48)	< 0.001
COPD (n,%)	4,961 (24.45)	484 (18.98)	351 (17.57)	88 (19.13)	< 0.001
Diabetes (n,%)	2,669 (13.15)	263 (10.31)	229 (11.14)	55 (11.96)	< 0.001
Cancer (n,%)	632 (3.11)	64 (2.51)	45 (2.19)	14 (3.04)	0.053
Parkinson disease (n,%)	1,229 (6.06)	402 (15.76)	64 (3.11)	22 (4.78)	< 0.001
Fractures (n,%)	1,679 (8.27)	185 (7.25)	167 (8.13)	41 (8.91)	0.323
Charlson index, median (IQR)	5 (4, 6)	5 (5, 6)	5 (5, 6)	5 (5, 6)	< 0.001
Exposure at admission (n, %)	-	2,226 (87.3)	1,747 (85.0)	429 (93.3)	< 0.001
Exposure time in months, median (IQR)	-	7.7 (2.8, 19.5)	8.4 (2.8, 19.4)	7.1 (2.6, 17.4)	0.201
Exposure time < 30 days (n, %)	-	346 (13.6)	249 (12.1)	69 (15.0)	0.158
Follow-up time in months, median (IQR)	16.5 (6.3, 33.0)	19.3 (5.8, 37.5)	17.8 (6.1, 36.2)	22.8 (8.1, 43.2)	< 0.001

GIR: groupe iso-ressources, according to the French national tool AGIRR for dependency assessment. MMSE: Mini Mental Status Examination. COPD: chronic obstructive pulmonary disease

**Fig. 1:**
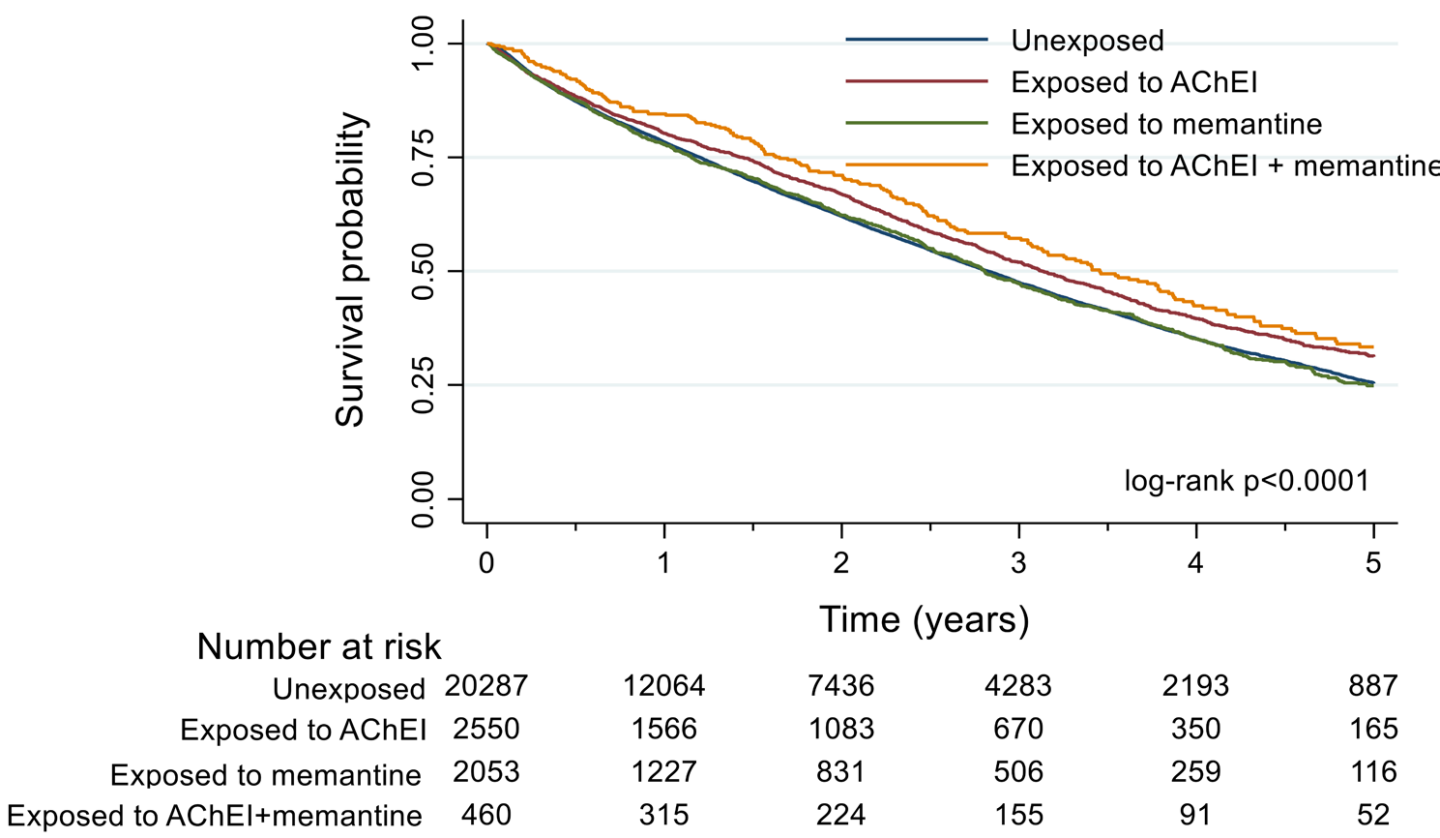
Survival plots for residents exposed or not exposed to acetylcholinesterase inhibitors (AChEI), memantine or AChEI plus memantine in the whole cohort

As compared to unexposed residents, unadjusted hazard ratios for mortality were significantly reduced in residents exposed to AChEI and in those exposed to both AChEI and memantine but not in those exposed to memantine alone. The survival plot is shown in Fig. 1. Adjusted hazard ratios for mortality were significantly reduced for the three groups exposed to anti-dementia drugs, as compared to reference group with two models that account for comorbidities differently (Table 2). The adjusted hazard ratio for combined therapy (AChEI plus memantine) was not significantly different from that for AChEI alone or memantine alone (*p* = 0.191 and *p* = 0.083, respectively). The one-year mortality rate was significantly reduced in residents exposed to the AChEI alone and those exposed to AChEI plus memantine (Supplementary material, Table S2). A sensitivity analysis was performed by excluding the 664 residents whose duration of exposure to anti-dementia drugs was less than 30 days and adjusted hazard ratios showed consistent results with those obtained in the entire cohort (Supplementary material, Table S1).

**Table 2:** Unadjusted and adjusted mortality hazard ratio and their 95% confidence interval for exposure to anti-dementia drugs among nursing home residents with dementia (entire cohort)

Exposure to anti-dementia drugs	Hazard ratio	95%CI	*P*
	**Unadjusted**		
None (reference)			
Cholinesterase inhibitors only	0.874	(0.821 to 0.929)	< 0.001
Memantine only	1.002	(0.938 to 1.069)	0.955
Cholinesterase inhibitors plus memantine	0.784	(0.686 to 0.897)	< 0.001
	Adjusted (model 1)*		
None (reference)			
Cholinesterase inhibitors only	0.826	(0.769 to 0.888)	< 0.001
Memantine only	0.857	(0.795 to 0.923)	< 0.001
Cholinesterase inhibitors plus memantine	0.742	(0.640 to 0.861)	< 0.001
	Adjusted (model 2)**		
None (reference)			
Cholinesterase inhibitors only	0.830	(0.772 to 0.892)	< 0.001
Memantine only	0.877	(0.813 to 0.946)	< 0.001
Cholinesterase inhibitors plus memantine	0.762	(0.657 to 0.885)	< 0.001

*adjusted on age, sex, level of dependency, Mini Mental Status Examination score, Charlson index

**adjusted on age, sex, level of dependency, Mini Mental Status Examination score, cardiovascular disease, peripheral arterial disease, chronic obstructive pulmonary disease, diabetes and cancer

### Propensity-score matched cohorts

**Fig. 2:**
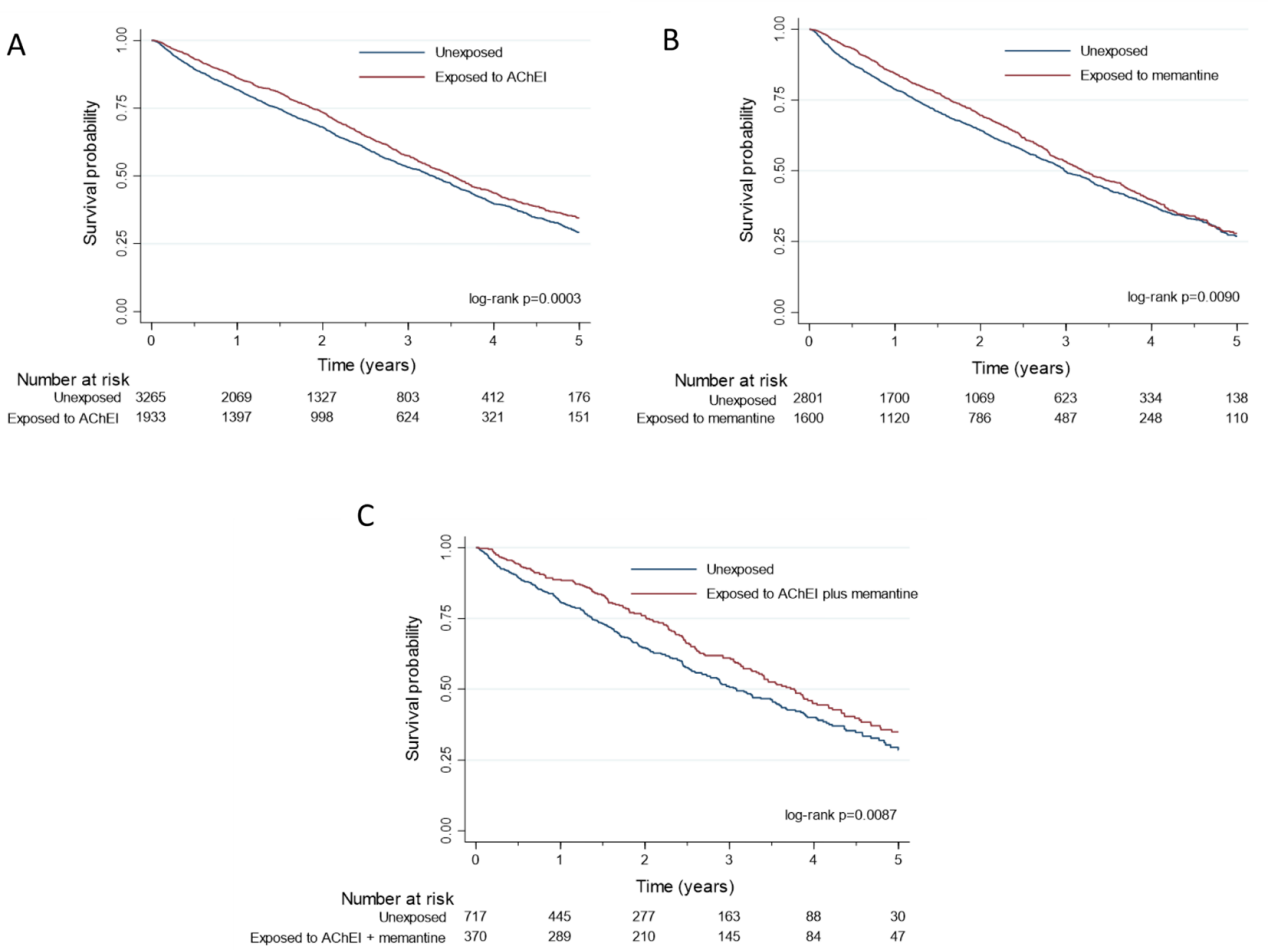
Survival plots for residents exposed or not exposed to dementia drugs in propensity score-matched cohorts. Panel A shows the survival plot for residents exposed to acetylcholinesterase inhibitors (AChEI) and residents not exposed to dementia drugs (matched cohort 1). Panel B shows the corresponding plot as a function of memantine exposure (matched cohort 2) and panel C as a function of AChEI and memantine exposure (matched cohort 3)

Three cohorts, each composed of residents exposed to one type of anti-dementia drug regimens and of residents not exposed to anti-dementia drugs, were formed using propensity score matching on age, sex, level of dependence, MMSE score and Charlson index values. The first cohort was formed of 1933 residents exposed to AChEI alone and 3226 unexposed to anti-dementia drugs, the second of 1600 residents exposed to memantine alone and 2801 unexposed to anti-dementia drugs, and the third of 370 exposed to both AChEI and memantine and 717 unexposed to any anti-dementia drugs. The characteristics of the residents selected for matched cohorts are presented in Table 3. For the three cohorts, the mean standardized differences for the matching variables were less than 0.20 (Supplementary material, Figure S2), indicating a satisfactory balance. Exposure to anti-dementia drugs was associated with significantly lower mortality in all three matched cohorts, with unadjusted and adjusted hazard ratios fairly close to that observed in the entire cohort (Table 4). To facilitate an overall view of the reduction in mortality associated with exposure to anti-dementia drugs, we have summarised the hazard ratios obtained in the different cohorts in a single table. (Supplementary material, Table S3).The survival plots for the three matched cohorts shows that mortality is significantly reduced throughout the follow-up period (Fig. 2). One-year mortality rate in the three cohorts was significantly reduced in residents exposed to the anti-dementia drugs (Supplementary material, Table S2). To estimate the time afforded by the reduction in mortality associated with anti-dementia drugs, we calculated the mean differences in survival time between exposure groups for the residents who died during the observation period. The survival time was 3.33 months longer on average with AChEI (first matched cohort), and the corresponding were 4.65 and 8.88 months for memantine alone and AChEI plus memantine respectively (second and third matched cohorts).

**Table 3:** Characteristics of the residents selected into the three cohorts matched on propensity scores. Cohort 1, 2 and 3 comprised residents exposed to acetylcholinesterase inhibitors (AChEI) only, memantine only, or AChEI plus memantine, respectively, and residents unexposed to anti-dementia drugs

	Matched Cohort 1			Matched Cohort 2			Matched Cohort 3		
	None	AChEI	*P*	None	Memantine	*P*	None	AChEI plus memantine	*P*
	(*n* = 3226)	(*n* = 1933)		(*n* = 2801)	(*n* = 1600)		(*n* = 717)	(*n* = 370)	
Age, years (m, sd)	84.8 (7.38)	84.4 (6.42)	0.027	85.6 (7.39)	85.2 (6.24)	0.050	83.9 (7.87)	83.5 (6.56)	0.358
Males (n,%)	1,044 (31.97)	602 (31.14)	0.558	856 (30.56)	494 (30.88)	0.838	242 (33.75)	117 (31.62)	0.497
Level of dependency (n,%)			0.840			0.658			0.640
very severe (GIR1)	176 (5.39)	109 (5.64)		183 (6.53)	124 (7.75)		68 (9.48)	34 (9.19)	
severe (GIR2)	1,279 (39.16)	754 (39.01)		1,209 (43.16)	671 (41.94)		362 (50.49)	167 (45.14)	
moderately severe (GIR3)	793 (24.28)	443 (22.92)		688 (24.56)	384 (24.00)		123 (17.15)	71 (19.19)	
moderate (GIR4)	779 (23.85)	487 (25.19)		549 (19.60)	324 (20.25)		132 (18.41)	78 (21.08)	
mild (GIR5)	138 (4.23)	79 (4.09)		125 (4.46)	67 (4.19)		18 (2.51)	11 (2.97)	
none (GIR6)	101 (3.09)	61 (3.16)		47 (1.68)	30 (1.88)		14 (1.95)	9 (2.43)	
MMSE score (m, sd)	17.0 (7.29)	17.3 (8.62)	0.195	16.1 (7.44)	16.2 (9.50)	0.794	15.2 (7.73)	15.5 (9.68)	0.572
Charlson index (median, IQR)	5 (4, 6)	5 (4, 6)	0.779	5 (5, 6)	5 (5, 6)	0.710	5 (4, 6)	5 (5, 6)	0.241

GIR: groupe iso-ressources, according to the French national tool AGIRR for dependency assessment; MMSE: Mini mental status examination

**Table 4:** Unadjusted and adjusted mortality hazard ratios and their 95% confidence intervals as a function of dementia drug exposure in the three matched cohorts. For each cohort, the hazard ratio was calculated using residents not exposed to anti-dementia drugs in the same cohort as a reference

Exposure to anti-dementia drugs	Hazard ratio	95%CI	*P*
	**Unadjusted**		
In matched cohort 1			
Cholinesterase inhibitors only	0.853	(0.783 to 0.930)	< 0.001
**In matched cohort 2**			
Memantine only	0.887	(0.810 to 0.970)	0.009
**In matched cohort 3**			
Cholinesterase inhibitors plus memantine	0.780	(0.648 to 0.940)	0.009
	Adjusted*		
**In matched cohort 1**			
Cholinesterase inhibitors only	0.870	(0.799 to 0.949)	0.002
**In matched cohort 2**			
Memantine only	0.882	(0.806 to 0.966)	0.007
**In matched cohort 3**			
Cholinesterase inhibitors plus memantine	0.778	(0.643 to 0.936)	0.008

*adjusted on age, sex, level of dependency, Mini Mental Status Examination score, Charlson index

## Discussion

This observational study found the use of AChEI, memantine or their combination is associated to a significantly lower all-cause mortality in a large population of nursing home residents. Consistent findings were obtained in the whole cohort and in propensity score-matched cohorts.

The beneficial association of memantine on mortality in nursing home residents with dementia is a novel finding, as no large-scale study had previously documented such an effect. Lazzeroni observed in a large database that patients treated with memantine had lower mortality than those treated with donepezil, but their study did not compare them with demented patients who had not received anti-dementia drugs [16]. Long-term follow-up of a randomized clinical trial of 75 patients with Lewy body dementia or Parkinson’s disease revealed that memantine was associated with better survival at 36 months [17]. Meta-analysis of randomized controlled trials on memantine in patients with Alzheimer’s disease have documented the beneficial effects of memantine on parameters other than mortality, in particular cognition and functional independence, and observational studies also suggest favorable effects on behavioral disturbances [18–20]. Although the effect size of memantine on the clinical consequences of dementia is considered small, all these results are consistent with ours.

Our results also confirm that the use of AChEI is associated with lower mortality in patients with dementia, a point highlighted in a recent meta-analysis of both randomized and observational studies [15, 21]. Among these meta-analyzed studies, only one had been conducted in nursing home residents with dementia, and it showed that the use of donepezil was associated with a reduction in mortality, with a hazard ratio of 0.89 (95% CI, 0.83–0.95) close to that observed in our study for AChEI [14]. In our study, we also observed that nursing home residents receiving combined therapy (AChEI plus memantine) had lower mortality that those with AChEI alone or memantine alone but AChEI differences in hazard ratio did not reach significance. Thus, our results suggest that combination therapy offers no clear benefit in terms of mortality compared to AChEI or memantine therapy alone. Studies that have compared the clinical effects of combined therapy with those of AChEI or memantine alone have come to divergent conclusions, and the superiority of combined therapy is still the subject of controversy [8, 13, 18, 22, 23].

One explanation for the reduction in all-cause mortality in residents exposed to AChEI could be linked to their cardiovascular effects. AChEI increase vagotonic tone and slow heart rate [11, 24–26], and resting heart rate has been shown to be an independent risk factor for cardiovascular and all-cause mortality [27]. These pharmacological properties may explain why AChEI is associated with lower cardiovascular mortality, as slowing heart rate appears to be a major component of the beneficial effects of several drugs on cardiovascular events [23]. Another possible explanation for our findings would be the metabolic effects of AChEI and memantine. Some studies have suggested that AChEI may have anti-oxidant properties, anti-inflammatory activity and a protective effect on endothelial cells, in patients with Alzheimer’s disease or metabolic syndrome [28–33]. Memantine antagonizes NMDA receptors which are widely distributed in the human body and might contribute to protect cells from excitotoxicity and cellular calcium excess [12]. By this way, it might have beneficial effects out of the nervous system, in particular on inflammation, cardiovascular diseases, cancer or infectious diseases. Those effects might also contribute to beneficial survival.

Another explanation would be a disease-modifier effect of AChEI, slowing progression of dementia and delaying decline in independence. A diagnosis of any type of dementia is associated with increased mortality, especially from pneumonia and neurologic causes, and dementia severity is correlated with the risk of dying [34–36]. In vitro studies suggest that AChEI may have a neuroprotective effect independent of its cholinergic activity [37, 38]. This hypothesis is further supported by the finding, in several studies, that dementia symptoms progressed more rapidly in patients in whom AChEI were discontinued than in patients who kept on treatment [39, 40], and that placement in nursing home was delayed in patients treated with AChEI [41–43].

This study has both limitations and strengths. We used data derived from routinely collected clinical records, which may contain errors, undereporting and missing information, particularly for the record of comorbidities and for the diagnosis of dementia and its type, both of which depend on the practice of the physicians caring for the residents. In particular, dementia is under-diagnosed or under-reported in such facilities, and when the diagnosis is recorded, the type of dementia is often missing. This is important because mortality rates differ between subtypes of dementia. In particular, vascular dementia, which has a higher mortality rate than Alzheimer’s disease [44], is probably more common in unexposed residents than in exposed ones, given that no anti-dementia drugs are indicated in this subtype of M-NCD. As in observational studies, our results may be influenced by biases, including unobserved confounders. In particular, the decision to prescribe dementia medication was not random, and we observed several differences in resident characteristics as a function of dementia medication exposure. We attempted to minimize this bias by conducting two different adjusted analyses, the results of which were found to be highly consistent. The strengths of our study are the large sample of residents distributed nationwide, the real-life context, the use of several relevant covariates known to influence mortality, with exhaustive follow-up and vital status record at the end of the observations.

While our study shows that the use of conventional anti-dementia drugs is associated with a reduction in resident mortality, it also highlights the fact that this population is largely undertreated by these drugs. This is not unique to our study, and has also been observed in nursing homes in the USA [45] and the Netherlands [46]. The reasons for this are not clearly known, and the relative frequency of subtypes of M-NCD for which these drugs are not indicated cannot explain it. This undertreatment may lead to a loss of chance for this complex and vulnerable population. At a time when anti-amyloid monoclonal antibodies have been approved in the USA to treat Alzheimer’s disease, our study is a reminder that the use of conventional Alzheimer’s drugs is important, particularly in patients living in geriatric institutions who are highly vulnerable and who will not be good candidates for these new treatments.

### Electronic supplementary material

Below is the link to the electronic supplementary material.

Supplementary Material 1

## Data Availability

The datasets generated and/or analyzed as part of this study are not available to the public, as the third party who supplied them has not authorized their sharing.

## Abbreviations

NH: Nursing home
AChEI: Cholinesterase inhibitors
EHR: Electronic health record
MMSE: Mini mental status examination
M-NCDs: Major neurocognitive disorders
