# Correction: Cholinesterase inhibitors and memantine are associated with a reduced mortality in nursing home residents with dementia: a longitudinal observational study

**DOI:** 10.1186/s13195-024-01548-y

**Published:** 2024-08-05

**Authors:** Charlotte Havreng-Théry, Bruno Oquendo, Victoria Zolnowski-Kolp, Pierre Krolak-Salmon, François Bertin-Hugault, Carmelo Lafuente-Lafuente, Joël Belmin

**Affiliations:** 1 Laboratoire LIMICS, Sorbonne Université, Paris, France; 2 Service de Gériatrie, Hôpital Charles Foix, Ivry-sur-Seine, 94200 France; 3 Présage Care, Paris, France; 4 Groupe Orpéa, Puteaux, France; 5 Laboratoire CEPIA, Université Paris Est, Créteil, France

**Correction: Alz Res Therapy16**,** 117 (2024)**

10.1186/s13195-024-01481-0

Following publication of the original article [1], the authors corrected a typographical error in the Abstract and Methods section. The number of nursing homes was 229, not 329.

The original article [1] has been updated.
