# Supplementary material for: Cholinesterase inhibitors and memantine are associated with a reduced mortality in nursing home residents with dementia: a longitudinal observational study
Source: Alzheimers Res Ther. 2024 May 29;16:117. doi: 10.1186/s13195-024-01481-0 (PMC11134888; doi:10.1186/s13195-024-01481-0)
Supplement: Supplementary file 1 — Supplementary Material 1 [file 13195_2024_1481_MOESM1_ESM.docx]

**Supplementary material:**

**Cholinesterase inhibitors and memantine are associated with a reduced mortality in nursing home residents with dementia: a longitudinal observation study.**

**Havreng-Théry C, et al.**

Table S1: Adjusted mortality hazard ratio and its 95% confidence interval for anti-dementia drug exposure in nursing home residents with dementia who were exposed for more than 30 days versus those who were not exposed to anti-dementia drugs (sensitivity analysis).

| **Exposure to anti-dementia drugs** | **Hazard ratio** | **95%CI** | **P** |
| --- | --- | --- | --- |
| None (reference) |  |  |  |
| Cholinesterase inhibitors only | 0.789 | (0.732 to 0.852) | <0.001 |
| Memantine only | 0.832 | (0.770 to 0.899) | <0.001 |
| Cholinesterase inhibitors + memantine | 0.725 | (0.619 to 0.850) | <0.001 |

Table S2 : One-year mortality (n and %) among nursing home resident according to exposure to to anti-dementia drugs in the entire cohort and in the three propensity score matched cohorts.

|  | **Exposure to anti-dementia drugs** | | | | **P** |
| --- | --- | --- | --- | --- | --- |
|  | None | AChEI | Memantine | AChEI+memantine |  |
| Entire cohort (N) | 20,293 | 2,550 | 2,055 | 460 |  |
| Deaths (n, %) | 3,875 (19.1) | 426 (16.9) | 390 (19.2) | 60 (13.4) | <0.001 |
| Matched cohort 1 (N) | 3,266 | 1,933 | - |  |  |
| Deaths (n, %) | 528 (16.2) | 243 (12.6) |  |  | <0.001 |
| Matched cohort 2 (N) | 2,801 | - | 1,600 | - |  |
| Deaths (n, %) | 530 (18.9) |  | 226 (14.1) |  | <0.001 |
| Matched cohort (N) | 717 | - | - | 370 |  |
| Deaths (n, %) | 123 (17.2) |  |  | 39 (14.9) | <0.001 |

AChEI: acetylcholinesterase inhibitors

Table S3: Summary of adjusted hazard ratio for mortality in nursing home residents exposed or non exposed to anti-dementia drugs in the entire cohort and in the three propensity score matched cohorts.

| Exposure | Entire cohort | | Matched cohorts | |
| --- | --- | --- | --- | --- |
|  | aHR | (95%CI) | aHR | (95%CI) |
| None | reference |  | reference |  |
| AChEI | 0.826 | (0.769 to 0.888) | 0.853 | (0.783 to 0.930) |
| Memantine | 0.857 | (0.795 to 0.923) | 0.887 | (0.810 to 0.970) |
| AChEI plus memantine | 0.742 | (0.640 to 0.861) | 0.780 | (0.648 to 0.940) |

AChEI: acetylcholinesterase inhibitors


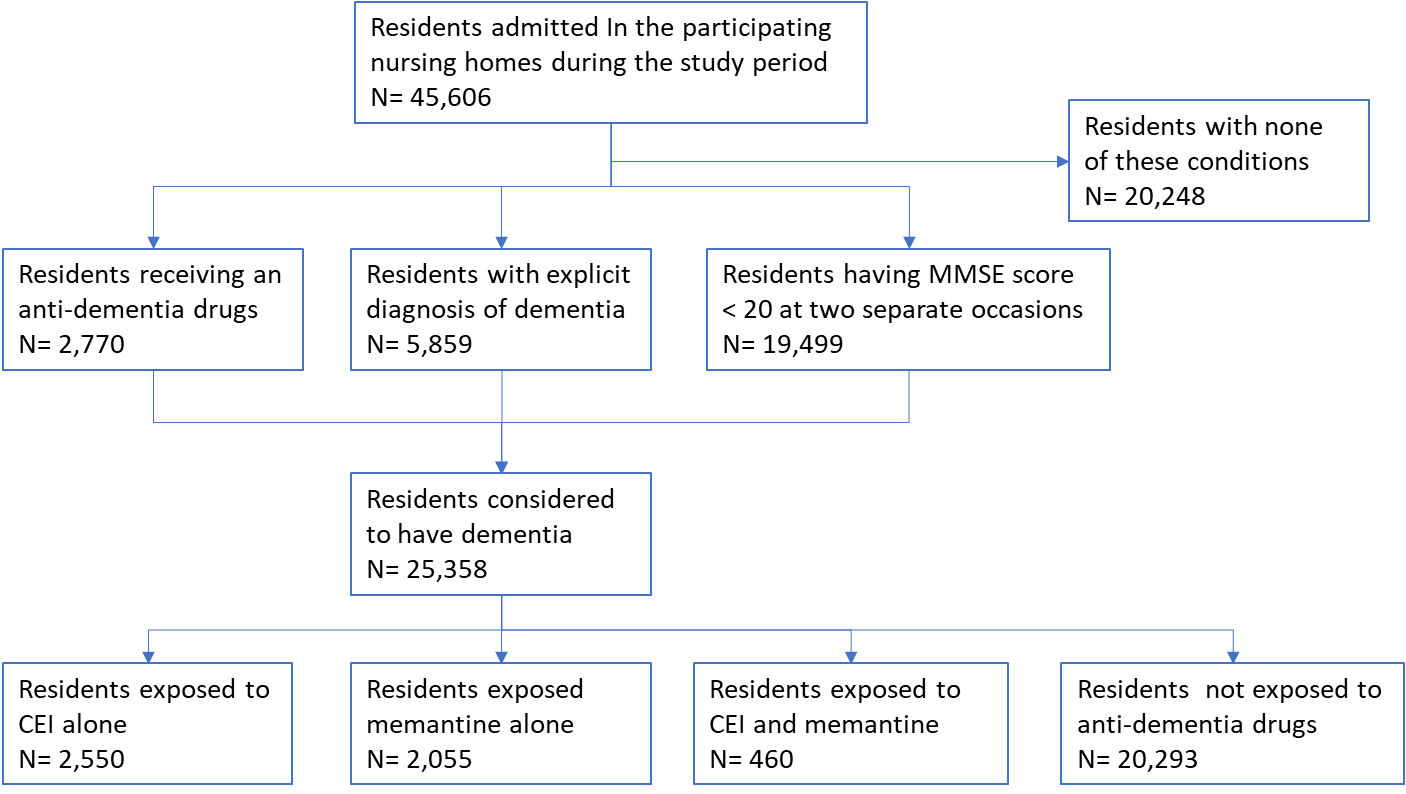


Figure S1: Flow chart of the study.


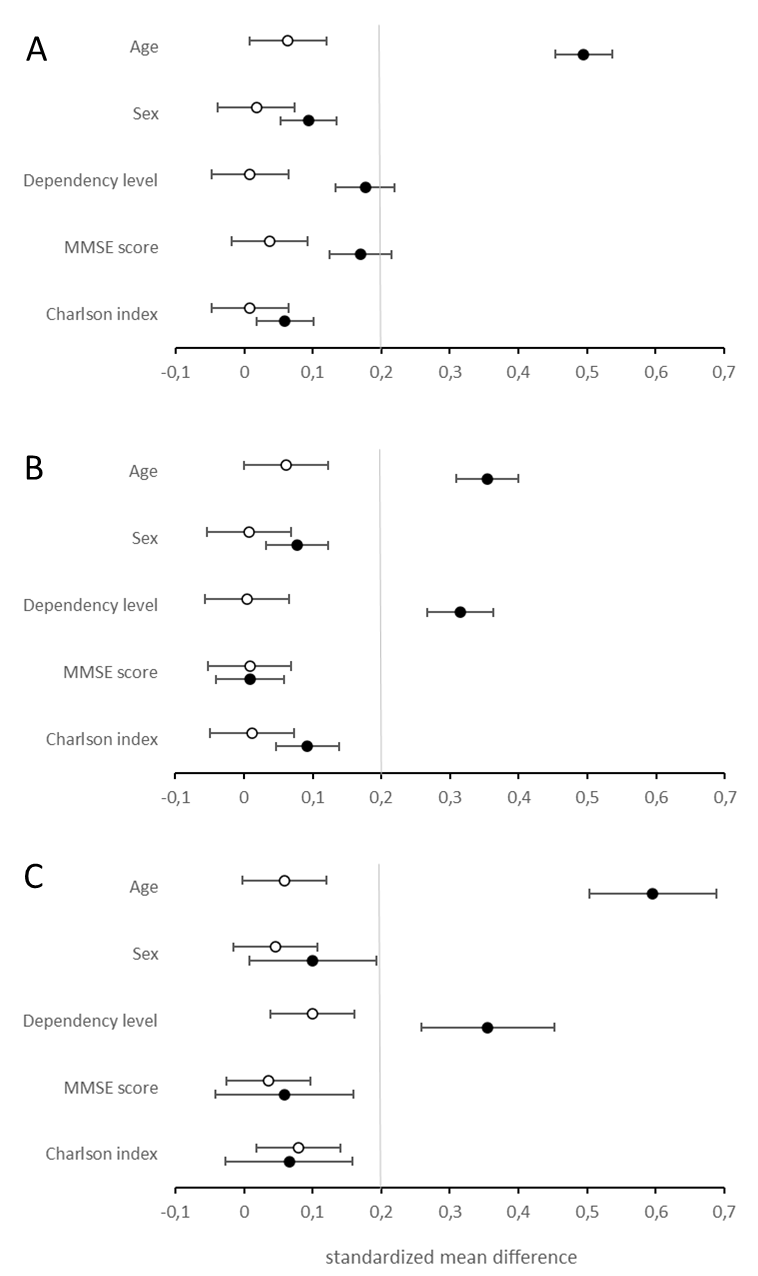


Figure S2: Standardized mean differences (SMDs) between residents exposed or not exposed to anti-dementia drugs for age, sex, level of dependency, MMSE score and Charlson index, and their 95% confidence intervals in the whole cohort (solid circles) and in the propensity score-matched cohorts (open circles). Panel A shows the SMDs between residents exposed to cholinesterase inhibitors and residents not exposed to dementia drugs. Panel B shows the corresponding SMDs as a function of memantine exposure and panel C as a function of CEI plus memantine exposure. A SMD greater than 0.20 was considered a sign of imbalance.
